# Editorial: Ecological management of invasive alien plants

**DOI:** 10.3389/fpls.2023.1345472

**Published:** 2023-12-05

**Authors:** David Roy Clements, Shicai Shen

**Affiliations:** ^1^ Department of Biology, Trinity Western University, Langley, BC, Canada; ^2^ Key Laboratory of Prevention and Control of Biological Invasions, Ministry of Agriculture and Rural Affairs of China, Agricultural Environment and Resource Research Institute, Yunnan Academy of Agricultural Sciences, Kunming, China; ^3^ Key Laboratory of Green Prevention and Control of Agricultural Transboundary Pests of Yunnan Province, Agricultural Environment and Resource Research Institute, Yunnan Academy of Agricultural Sciences, Kunming, China

**Keywords:** resilient ecosystems, competitive crops, physical environment, climate change, succession, evolution, agroecology

Invasive alien plants continue to spread across the globe, and the associated economic and ecological costs are also rising. Since the industrial revolution, globalization has promoted the spread of invasive alien plants through the movement of people and goods. Climate change is also exacerbating the spread of invasive plants. In September 2023, The Intergovernmental Science-Policy Platform on Biodiversity & Ecosystem Services (IPBES) released the first ever *Assessment on Invasive Species and Their Control*. This comprehensive report calls for urgent action to mitigate increasing threats by invasive species seen in every world region.

Since the widespread adoption of pesticides in the 1940s, management of invasive plants has often prioritized pesticides as control tools. Given high costs, resistance development, and negative environmental impacts of herbicides, there is much interest in ecological management methods. The ultimate goal of ecological management is to produce ecologically resilient plant communities. In their native environments, populations of invasive alien plants tend to be regulated biotically by natural enemies or competitors. Ecological management aims to replicate such population regulation in the invaded environment.

We encouraged researchers to contribute to our Research Topic on ecological management of invasive alien plants within relevant themes. The following five themes were featured among the contributions: 1) ecological methods to create more diverse and resilient ecosystems, 2) utilizing competitive crops or native plant species to outcompete invasive alien plants, 3) manipulation of the physical environment to help manage alien invasive plants, 4) ecological approaches to slow the spread of invasive alien plants enhanced by climate change, and 5) long-term management approaches to invasive alien plant management in view of long-term successional and evolutionary processes.

Under the first theme on developing more diverse and resilient ecosystems, the contribution by Tataridas et al. highlight the “winner-takes-all” contest between invasive alien plants and agroecology. They explain how agroecology must employ ecological principles to devise resilient, diversified, and sustainable systems. These ideal agroecological characteristics contrast with the tendency of invasive plants to exhibit dominance, reduce biodiversity, and degrade ecosystems. The authors describe successful examples where agroecosystems have incorporated more diversity, such as crop diversification or legume-based crop rotations, and the use of citizen science to detect incipient invaders. Another contribution to the same theme was by Gong et al. The invasive species they studied is one of the most notorious global invaders, bamboo – specifically *Phyllostachys edulis*. Although invasion by *P. edulis* did reduce the density of broadleaf trees in their study, the trees that survived exhibited greater radial growth than in uninvaded forests, demonstrating resilience in these forest ecosystems.

A more diversified ecosystem also incorporates the second theme of utilizing competitive crops, as researched by Shen et al. Their contribution to the Research Topic examined the ramifications of using two different competitive crops to reduce population of the invasive alien plant, *Mikania micrantha* (mile-a-minute). Previous work had shown strong competitive effects of sweet potato (*Ipomoea batatas*) on mile-a-minute, but the combination of sweet potato and hyacinth bean (*Lablab purpureus*) reduced growth of mile-a-minute even more. Physiological differences between the two crops, including differences in soil nutrient utilization, acted in complementary fashion to reduce the niche space available to mile-a-minute.

Better knowledge of the utilization of nutrients by invasive alien plants in comparison to native plants can help with the management of the invasives through manipulation of the physical environment. In their contribution, Guan et al. demonstrated very different N use patterns by the invasive *Solidago canadensis* than co-occurring native plant, *Artemisia lavandulaefolia*. The researchers utilized four sites, with 3 habitat types within each: farmland, wasteland, and roadside. This allowed them to test how effectively the two species acquired N in different forms, with the greater plasticity of N acquisition by *S. canadensis* explaining much of its success across a variety of habitats.

Two contributions to the Research Topic examined how our understanding of invasive plant ecology must reckon with climate change. Huang et al. looked at climate change impact on two *Erigeron* weed species. They predicted that climate change will allow the very adaptable *E. annuus* to expand its range considerably in China, whereas the expansion potential for *E. philadelphicus* is more limited. Yang et al. also looked at two invasive species likely to expand their distributions under climate change in China, *Lolium temulentum* and *Aegilops tauschii*. At present, habitat suitability models showed considerably more habitat is available for *A. tauschii*, but under climate change scenarios suitable habitat will increase greatly for both species. Such potential range expansion needs to be monitored vigilantly because both species may cause severe economic damage in wheat crops, *A. tauschii* exhibits high levels of herbicide resistance, and *L. temulentum* is poisonous to humans and livestock.

Finally, two contributions addressed the theme of ecological management in light of long-term successional and evolutionary processes. Malka et al. examined seed germination characteristics among different populations of *Parthenium hysterophorus*. Though this weed is a serious invasive issue in some 50 countries worldwide, much of its reproductive and genetic potential is not well characterized. Among five populations, the researchers identified variation in seed size and weight, and germination characteristics, and also demonstrated potential transgenerational shifts. Zhao et al. investigated environmental factors affecting invasion by *Ambrosia artemisiifolia*. Their contribution helps to understand the important influence of habitat on the probability of invasion. To establish *A. artemisiifolia* populations, only 60 seeds m^-2^ were required in roadside habitats, whereas forests required 398 seeds m^-2^. The presence of resilient ecosystems with a large native plant component clearly reduced the probability of invasion by *A. artemisiifolia*, as did habitats below the plant’s available moisture threshold.

These contributions to the Research Topic largely focused on improved understanding of the ecology of alien invasive plants, rather than concrete applications of ecological knowledge to management. This very much reflects the current state of the field, with much basic ecological research still required, as well as “proof-of-concept” research utilizing field trials of ecological management. However, these ecological approaches are urgently required, and thus it behooves us to make more rapid progress in this critical field.

## Author contributions

DC: Conceptualization, Writing – original draft, Writing – review & editing. SS: Conceptualization, Writing – review & editing.

